# Malaria, and Its Connection with the Existence and Character of Disease

**Published:** 1850-05

**Authors:** Samuel Thompson

**Affiliations:** Albion, Ill.


### ARTICLE I.

*Malaria and its Relations to the Existence and Character of Disease. A Paper read before the Æsculapean Society.*—By SAMUEL THOMPSON, M.D., of Albion, Ill.

I have taken for the subject of the present paper, a rather wide subject, and perhaps shall find few who will coincide in the views I shall advance. They are, however, the result of much reflection and considerable observation, and to placing them in their present form, I have been led by the perusal of a long list of notes of cases, occurring in different localities, at different seasons, and in different years, as well as by the fact that the question to some extent involves the ætiology of at least one disease, to which popular opinion attributes a special cause, and the proving which to depend on malarious origin, would certainly be a public benefit. At the same time, to show the coincident action of malaria, may perhaps relieve us from much fear and a good deal of perplexity in relation to apparent danger from contagion, in cases where the simultaneous or successive invasion of a disease on many persons, gives rise to suspicion.

Previous, however, to pointing out the effects of malaria, it may be as well to say some little in reference to its existence, as some writers of reputation, among whom Dr. Bell, of Philadelphia stands prominent, deny that there is any such thing, and consider that all those effects usually ascribed to its influence, can be better explained by reference to common causes, such as vicissitudes of heat and moisture; and regard the belief in malaria as visionary and unphilosophical. But with the highest respect for the Doctor and his supporters, I must demur to such views, considering that it is much less a stretch of imagination, to believe that the atmosphere may be the vehicle for conveying the cause of certain symptoms and appearances, when we know that effects at any rate analagous may be artificially induced by certain agents inhaled with the air, than to invoke the power of vicissitudes of either Thermometer or Hygrometer to explain, what such alternations alone (how popular soever the idea) are not proved to produce.

That there is no such connection between atmospheric vicissitudes, and the peculiar character of disease, will, I think, clearly appear, from the following facts, derived from Dr. Torrey's able statistical work on the climate of the United States. I will refer to the records of three stations only.

At Fort Gratiot, latitude 43 North. The general sickness is Fever and Ague. The river on which it is situated is bordered by broad marshes, where also the Cholera in 1832 raged most violently among the troops. Here the range of the thermometer in the year is about 110°.

At Fort Brady, in latitude 46.39,—with same range of thermometer. The location on the south bank of the St. Marie. To the S.W., beyond some cultivated lands, runs a marsh. But the prevailing winds are from W., N.W., and S.E.—the latter passing over the marsh,—the former a mountainous country; yet here Intermittents and Remittents are rare; Inflammatory diseases and Typhus being most common. (It is a pity there is no statement of the relations of the invasions of Typhus with the previously existing wind, as McIntosh, Armstrong, and others, consider Typhus a disease of malarious origin.) But it is certainly a much more healthy post than the former. Here vicissitudes are well matched, and the diseases, if resulting from them, should be of the same description.

At Fort Trumbull, latitude 41, near Long Island Sound, where the mean range of the thermometer is 78.° Malarious diseases, as they are commonly called, are unknown; while in Florida, where the range of the thermometer is but a few degrees, Intermittent and Remittent Fevers abound. Indeed, were this doctrine of vicissitudes correct, the climate of Florida should be among the most healthy in the world—the mean range of the thermometer at Key West being only 37°; yet it is known to be the most unhealthy of all the Florida stations: while at Fort King, where, from its inland situation, the range of the thermometer is as much as 78°, the sickness is but trifling, at the same time, that from its secondary basis and swampy neighborhood its fevers are chiefly intermittent. Now the unhealthiness of localities, should on this principle increase just in proportion as they are more remote from large bodies of water ,as the sudden alternations are generally just in proportion to such remoteness. Yet we know that this is anything but true ; the difference in the salubrity of a place, as well as the character of the diseases, appearing generally to be associated with certain geological formations. Those lying upon primary rocks and covered by a poor sandy soil, being free from Malarious diseases, as in most parts of New England: while in the valley of the Connecticut, lying upon a secondary formation, like our valley of the Mississippi, and covered with a soil full of organic remains, they are indigenous: thus in the midst of a non malarious region, forming a striking illustration of the connection of such soil and substrata with Intermittent fever.

That vicissitudes of heat and moisture are quite inadequate to account for a certain class of diseases is manifest. For as Dr. Torry remarks that, "as the regions of New England, New Brunswick, and Nova Scotia are exempt from Intermittent, while in the region of the great Lakes, both on the British and American sides, it is very prevalent; and as the coast of the former exhibits climatic features similar to the other, so far as regards temperature and humidity, it follows that a solution of the question, must be sought in the admixture of terrestrial emanations dissolved or suspended in atmospheric moisture." That vicissitudes of heat and moisture are therefore quite inadequate to account for a certain class of diseases is manifest. Whether we can as clearly show any other causes is another question. It is however evident that there is some cause connected with locality. We all know that in a great majority of cases, moisture is essential to the production of this cause, and yet that excess of moisture is uncongenial to it also, as no one ever suspected pure and deep water of developing malaria, while few doubt the dangers of lands that have been submerged, but which are in a drying state; and who is there whose olfactory nerves have not told him, in a night ride in summer or autumn, that the air contained different elements in different spots?

But it is objected that Chemistry, that inspector of nature, can detect no change in the atmosphere in those spots said to be loaded with miasm. But I would ask, can Chemistry detect the particles exhaled by a grain of musk, and perfuming a whole room? Can Chemistry isolate the contagious exhalation from a small pox patient? Or, can she or the microscope detect the myriad germs of the Fungi? Yet we know these must exist and in their larger varieties we can see them, and who doubts that Musk or the blossom of the rose perfume the air, or that such perfume is indeed matter—part and parcel of that from which emanates.

But, say the anti-malaria men, you attribute this occult and mystical cause to the decomposition of vegetable substances. How then will you account for its manifestation in places such as the Spanish Sierra's (as noted by Fergusson) where no vegetation exists or can exist, in the almost dry beds of mountain torrents. But allowing all this—giving the fullest force to every point—allowing if you please that the waters of these torrents never swept a foot of land bearing vegetation, still what does it prove? Do I, does any one pretend to say, that Malaria, though probably generally emanating from vegetable decay, is vapor sui generis, and not formed of nature's common elements, Oxygen, Hydrogen, Nitrogen, or Carbon, or their, or other elementary combinations ; and who is there in this day shall deny that vegetable and inorganic matter may and will, in decomposition yield many of the same elements ? But is it true that Chemistry has really come to fault ? Can she indeed with her argus eye detect no trace of the foe. No bended grass, no ruffled leaf, to guide us to his lair ? No indeed ! some at least, of her scouts declare that they have found a trail, and though it be crossed and disturbed in spots, they say (perhaps with too sanguine faith) that they can detect his foot print among the fresh leaves, on the slimy marsh, in the slaty clay, aye, even by the cool spring side. Perchance they may have only found one of the *family*, but they have at any rate it appears, proved, that that one leaves a trace behind. Let us hear what they say. For years past it has been known that the copper sheathing of ships traversing certain waters, decayed with unusual quickness. To ascertain the cause of this great commercial evil, the Lords of the Admiralty of Great Britain, directed that some of the water from the mouth of the Gambia on the Coast of Africa, one of the spots where copper was so affected, should be carefully secured in glass bottles and sealed up, brought to England and submitted to the examination of that able chemist, Professor Daniell. In it he detected Sulphureted Hydrogen in large proportions. We all know that the coast of Africa is one of the most fatal places in the world to human life. A single night ashore often being supposed to destroy life, by developing a fever nearly akin to yellow fever or to our malignant congestives. Previous, however, to this, Dr. Malcolmson had suggested that the presence of this gas, which he had detected in the waters of the Saline lakes of different parts of the world, must arise from the decomposition of the Sulphates in the water by the carbonaceous matter of vegetables floating in them. Acting upon this idea Professor D. on the 2nd Nov. 1840, instituted the following experiments. He placed a quantity of newly fallen leaves in three glass Jars, capable of holding a gallon and a half of water each :

Upon the first he poured a gallon of river water.

Upon the 2nd the same quantity of water with one oz. of common salt.

Upon the 3rd the same quantity of water with one oz. of Sulph. Sodæ.

The three jars were then plaeed in a warm room, the temperature of which varied from 70 to 110, and the water was filled up from time to time and well stirred. Upon examining them on 5th Feb., 1841, (three months) the following was found to be the state of the jars. No. 1 had a very disagreeable odor, but produced no change whatever upon paper soaked in acetate of lead. No. 2 was perfectly sweet, and possessed indeed a rather agreeable odor. It produced an effect of course upon the test paper. No. 3 had a most insupportably sickening odor, much worse than that of pure sulphureted Hydrogen, and instantly blackened paper soaked in acetate of lead, throwing down sulphuret of lead with a metalic lustre.

Two gentlemen (Saerd and Oldfield) entrusted with conducting an expedition up the Niger, remarked upon the deadly effect of breathing the air of " Those accursed swamps, where one is oppressed, not only bodily but mentally, with an indescribable feeling of heaviness, langour, nausea and disgust ;" and such is the united evidence in relation to all spots where sea water comes in contact with vegetable matters.

Now, it has been experimentally proved by Thenard and Dupuytren, that so small a quantity as 1-1500th part of Sulphureted Hydrogen, dispersed in atmospheric air, kills small animals which breathe it, and the effect produced upon human beings inhaling it, are those very sensations of langour, heaviness and nausea, described above. But is this all that can be adduced in favor of the opinion that the nature of Malaria is gaseous, and that gas probably, sulphureted Hydrogen? No indeed! I think it was in 1845 that Dr. Gardner, Professor of Chemistry in Hampden Sydney College, in a paper, published in the American Journal of Medical Sciences, assuming that Sulph't. Hydrogen, was the active agent in the production of Malarious diseases on the coast, undertook the following experiments with a view to ascertain whether the same agent could be detected in those inland spots where periodic fevers also prevailed, and where Malaria was generally supposed to exist. Finding it impossible to procure a sufficient quantity of atmospheric air to test its presence, he determined to apply pure silver (one of the most delicate tests for Sulphur) as the means of trying the question. The purity of the silver he used, and the delicacy of the test was shown, by putting one drop of hydro-sulphate of Ammonia into 120,000 grains of water: a 5 cent piece placed in it was dissolved in a few minutes, proving that *pure* silver, is able to detect Sulphur, in a solution containing only one part in 3,000,000 parts of water. With silver thus prepared, he examined the air over various streams and marshes, and while in some of these it took weeks to produce any tangible effect, in others 22 hours were sufficient to render the presence of Sulphur evident, upon passing a stream of Hydrogen Gas over the pieces of coin when enclosed in a green glass tube and made red hot. Having thus proved that sulphur existed in such localities, his next object was to examine the source of it. In the foregoing experiments, the immediate source was a marsh containing much decaying vegetable matter; a rich alluvial soil saturated with spring water, or that which had percolated through the soil, and heated by the temperature of midsummer. Alluvial deposits contain much vegetable matter; their blackness greatly depends on it, and this vegetable matter is in a constant state of decay, the rapidity of which is proportioned to the access of oxygen and the warmth of the season. The second element of the sites in which Sulphureted Hydrogen was detected by Dr. Gardner, was decaying vegetation; in its decay carbon is left in excess and exerts all its powerful affinities to assume the gaseous form. But vegetables contain also certain inorganic constituents, which are of great interest in the present question. Of these, the Sulphates of Lime, Soda, Potassa, and Magnesia, have been selected; in the decay of a plant containing any of these salts, the result will depend upon the presence or absence of water. If dry, they will be unchanged, but if water and heat be present, the Sulphuric Acid will be decomposed. The leaf contains a large portion of the Salts existing in a plant; but of all agents, spring water is the most important—it is usually impure. It contains the soluble salts of the land through which it has percolated. These must, from the nature of the case, differ; Muriates, Sulphates, Phosphates, and Carbonates, have been found by different analysts. Spring water is seldom free from Sulphate of lime or magnesia; the former imparts to it the quality called hardness. When these ingredients are present in any quantity, and the water is kept in contact with decaying vegetable matter, they are decomposed; Oxygen is abstracted, and Sulphurets are produced; the latter in their turn yield Sulphureted Hydrogen, with the first nascent Hydrogen they encounter. So says Dr. Gardner: and when we compare these views and experiments with those of Professor Daniell, I conceive it will require powerful arguments and well collected facts to invalidate them. Sulphureted Hydrogen is a narcotic poison, prostrating the nervous system, and destroying muscular energy; in small quantities it produces colic and internal congestions. Well, still it will be objected perhaps, that so far all that we have said only proves that Sulphur exists in the water, in the soil and in vegetation, and that Hydrogen gas coming in contact with it gives rise to Sulphureted Hydrogen; but that we have have not proved that the existence of this gas is the cause of disease. To this, I reply—we have proved its existence in those places, especially where malarious diseases are most rife, and we have shewn that its effects, especially in minute quantities, are in many points similar to those we find as *symptoms* of *disease* peculiar to such regions. That, therefore, we are justified in coming to the conclusion, that like effects result from *like* causes, even should it turn out that they are not *identical* ones.

Dr. Bell asks why in large cities, where the sources of Sulphureted Hydrogen must be so plentiful, are not the effects of malaria more prevalent? To this I would answer,---that the *question* assumes the dispute, and that it is entirely an assumption to assert that its effects are not manifest. For, if as I believe, various diseases, even owning a common cause, do under different circumstances, manifest themselves in different grades or even forms, as resulting from the differences of circumstance, it must be evident to all, that inasmuch as cities are proverbially more unhealthy than the country, though the phases of disease are not always the same, it remains with the unbelievers in malarious causation, to show a more reasonable and efficient cause of such excess of disorder, than this gas which they admit exists there in such abundance.

But while I thus think it more than probable that Sulphureted Hydrogen plays a most important part in the ætiology of many of our diseases, I by no means intend to assume that it is absolutely and *alone* such cause. My arguments and quotations, have had one general aim,---viz: to prove the existence and agency of malaria, define it to be what you may; and to show that, not only can we prove its existence from theory reasoning, but that *it is*, and in one of its shapes, at least, *is tangible*. I disapprove of setting down malaria as a vision, and I equally object to hasty and positive conclusions as to its essence. I am too well aware. that in all investigations into the arcana of nature, we should walk with cautious steps, and enquiring eyes, conscious how often our apparent discoveries of truth, are but the shadows cast by some great fact we do not see or cannot grasp: or like those of the first voyagers in search of this continent may prove but some jutting headland or some stray island, parts of the whole, it is true, and if wisely and cautiously followed, invaluable as guides and incentives to further enterprize; but apt to frustrate all our hopes if we too eagerly conclude it to be the mainland, and consider our search ended. While perhaps, others more temperate, cautious and persevering, starting from our self satisfied halt, will reap the harvest and the honor, which were just within our grasp. Having thus far demonstrated, I conceive, that miasma is not a vision of a heated fancy, but that if we have not yet attained the key to its identity, we can at any rate, manifest that it has existence and location, I shall turn to the ultimate object of this paper. Namely: to endeavor to show that our diseases, though modified by season and locality, are generally children of one family, or at any rate, have some of the same blood flowing in their veins. In endeavoring to do this, I shall refer to a number of cases, grouping them wherever I can by a brief statement of their character and symptoms, and only occasionally detailing such as are necessary for illustration: and I would call attention to the merging of one class into the other, as seasons changed or localities varied, marking the fact that the basis, the wax was indeed the same, the chief difference being in the seal that marked its surface.

I shall commence with cases of what is called Milksickness; and the first I shall adduce occurred in the fall of 1846. The prominent symptoms were pain in the *head*, *back*, *legs*, and *elbows*. She had been sick for some time before, and for two days previous to being taken down, the bowels had been costive. The pulse during the whole continuance of the case, was about 100, weak and soft when she was quiet, but upon the least excitement or exertion becoming temporarily full and strong. There was obstinate constipation, incessant vomiting of acid matters, a sense of burning at the epigastrium. The skin was rather warm for the first two or three days, but after that, cool. The next case is of a somewhat similar character, occurring in Oct. 1847, and in a different neighborhood, but in a malarious district. (My notes are not full.)

W.B. *æt* about 45 years, had been ailing for some weeks, took some Calomel and oil, felt better afterwards, but never recovered his strength; said he felt pretty well, but could not work. On Wednesday 27th Oct., went outside his house to cut some sticks of firewood, but could not work above a few minutes without sitting down; could not eat his meals, and next day was much troubled with a sensation of burning at the epigastrium, which indeed, he had occasionally felt for weeks before. Had great eructations; no appetite; took some salts which operated from 7 A. M., till 7 P. M.; stools watery. I saw him on the 30th at 6 P.M., he had vomited twice this day, a quantity of bluish green water, with a thick, dark colored, grainy sediment; he could not eat and had great thirst; said he *felt* all right *below* the stomach; skin cool, feet had been cold and were still disposed to be so; pulse regular, about 80, a little sharp, but compressible; cheeks flushed; great nausea; had pain and soreness in upper maxilla, and occasionally in the hips, and over left side of the thorax, which were even tender to the touch; had a little cough and expectoration, but no pain on deep inspiration; no abnormal murmurs in lungs; the matter vomited was VERY sour; gave Soda Carb. but with no evident benefit; at 7 P. M. gave Quinine gr. x., Morphine gr. ¼, in pills; at 8 P. M., said he felt easier, but as if he had a knot in his stomach; left him with orders to take Quinine gr. v. Calomel gr. v. Morphine gr. ⅛, every 4 hours, with Soda Carb. ad libitum, for the vomiting, and Cremor Tart. Jalap and Soda, to keep the bowels in a solvent state. Saw him again next day; pulse then 80, and small, skin cold, still had the epigastric burning, had vomited excessively; a mistake was made in not giving him the calomel with the quinine; the bowels had been moved three times, but he had first to take all the powders, (comprising Cremor Tart. ℥. iv, and Jalap ℈j.) The motions were dark, watery and very offensive ; tongue moist, and rather red at the apex ; still had tenderness and burning at the epigastrium. Urine free ; cheeks flushed ; nausea and thirst excessive. Ordered cloths wrung out of cold water frequently changed to be applied to the epigastrium, with Calomel gr. vi. and Morphine gr. ¼. at the time, and Calomel gr. vi. and Jalap gr. viii. every 3 hours, till they operated ; give Soda water ad libitum, and if he should feel chilly, use sinapisms instead of the cold wet blankets. On Nov. 1st, he was better; no vomiting, pulse soft, 66, bowels freely moved, motions black and fœtid with some scybala; urine free ; no headache, (which had previously been present) has taken some chicken broth ; he kept gradually improving under the same course, with the addition ,towards the latter end, of Quinine and Iron and occasionally Turpentine. I dismissed the case on the 12th.

On 7th Oct. 1847, G. K. aged about 35, sanguine nervous temperament, was taken after very similar premonitory symptoms, with some little fever for the first two or three days, intense restlessness, incessant vomiting of sour matters with a sense of burning at the epigastrium ; *headache, pain in back and thighs ;* bowels costive, pulse oppressed, tongue pale. After the 2nd or 3rd day his skin became cool, but the vomiting continued. The bowels yielded to one or two active doses of ordinary purgative medicine, and the stools were like tar, and horribly offensive. He has since complained at various times, of threatenings of a return of the same, as he has languor, pains in the back, and limbs, &c., but he is a little fanciful I think.

On 18th Sept. 1847, I was called to attend the family of Mr. B., who had been deluded into putting himself under the tender mercies of one of the pepper-box fraternity, alias steamers. There were sick, Mr. B. and his wife, a little girl of 9 years, and a boy of 7 years old; the latter was dying of dysentery, and the tortures of red pepper injections which had been administered till the poor creature no longer possessed human intelligence, but upon each call of nature would bolt up in bed, and before any one could support him, fall headlong to the floor. He appeared to have lost all self government or consciousness beyond sensation in the rectum; that indeed, in his case, appeared to be the centre of sympathies. The others were well marked cases of the so called milksickness, occurring on a spot, on which it is—be its cause what it may—endemic. The symptoms were of course, not in their simple condition, as the violent and absurd means, which had been already employed, had of course, modified and exaggerated some of them very much. But the common symptoms of pain in the back, head and limbs; the intense craving thirst; the absence of any marked fever; the incessant vomiting of acid matters; the burning at the epigastrium; the obstinate constipation; the cold extremities; the restless indescribable distress; were all present; and in the husband, of a plethoric sanguine temperament, the over stimulation, and maltreatment had given rise to the most violent convulsions, with loss of consciousness, and a bounding pulse. The latter gave way to free v. s., All these patients had had the premonitory symptoms of languor and heavy limbs, popularly called the Tires. The treatment, though eclectic, was essentially the same, and all except the boy recovered. These cases were all, except the 2nd, on the west side of the county, towards the Little Wabash, where low swampy bottoms extended westward of them, and this circumstance applies equally to the second case, which occurred in the South part of the county. During the same period I find on my book brief minutes of a number of cases noted as irregular Intermittents, complicated with Dysentery, also occurring on the west side of the county.

In most seasons our most healthy district is to the east of the town, unless you go too near the Bonpas Creek. But from the beginning of July to the end of Aug. 1847, we had on the east, and extending north and south, a fever apparently different from what we are accustomed to. But I believe the difference was in grade only. It appeared to rage among those who lived in good houses and were well fed and clothed, as much as among those in different circumstances; (by one practitioner it was called a brain fever) and went *through* nearly all in a house when once it began. My notes supply me with few details, for I was too incessantly occupied to make many. But I would call it Typhus or Typhoid, (though I could discover no cutaneous rash or spots,) or rather as Armstrong & McIntosh call it, Congestive fever, and it tallied with Dr. M's. description most closely. The previous indisposition; the sense of oppression; the headache; the low, quiet delirium; the suffused conjunctiva; the teeth and lips covered with sordes, with the general tendency to Diarrhœa. and even in some cases to Dysentery; and be it observed, these symptoms only differ in degree, not in kind, from the group we have last described. It appeared to concentrate its greatest force on certain spots, and though many other cases occurred, they were more dispersed. Yet there was no ground to consider it contagious. In the house in which occurred the first case of it which I attended, the whole of those taken during the epidemic recovered; but the wife who had hitherto escaped, after nursing the whole family was suddenly taken with a congestive chill, and died in 12 hours; this was three weeks after I had quit attending the others, and just at the same time that the milksick and congestive character began to be most marked. But I look upon this case as of a kindred nature with the previous ones; only that it took a different shape from difference of constitution, and the exhausted state of the nervous system, from fatigue and anxiety. More north in the county, the cases took a more Dysenteric type. In the latter part of Aug. and Sept. the sickness changed a little, taking on a more intermittent, though not a milder form; approximating more to the character of the case I have just noted, though generally it was more prevalent towards the Little Wabash. I was called to one house on a bluff over the Little Wabash flats, where six were taken down in two days; some were immediately delirious with rending headache; others were cold and puking. Through October the sickness much abated, but still it maintained much the same outlines; and in many instances continued to bear the peculiar features of what is called milksickness. And I would request particular attention to the fact, that the time when the fevers in certain spots took the algid form, was also the time, when in other localities, the milksick group of symptoms became most prominent; in some cases the symptoms of each class, so running into one another, as to make the diagnosis a matter of difficulty. Through Nov. and Dec. the same typhoid tendency, which had so specially marked the middle months, gave a color to all the cases, modified as the weather grew colder, by Pneumonic complications, especially in the flat wet grounds, and in those who had been much exposed, or badly clothed; constituting what is so indefinitely called winter fever, or more properly Typhoid Pneumonia. We had at this time some mild cases of Scarlatina, in the town; but still, as previously, the chief sickness this year appeared to the west of the streams or flats. During a considerable part of the summer, we had a most unusual prevalence of easterly winds.

In regard to the early part of this year, the sickness was not of any particular character; scattering cases of Diarrhœa, Dysentery, Intermittent, &c., occurring, with, in June, two rather marked cases of typhoid fever. But as pointing out the generally progressive development of the prevailing type, already described, I find under the date of 9th June, this note upon my book. "J. H. and wife: disposition to drowsiness, when free from pain and headache; vertigo on stooping, or sudden motion; dark stools; a feeling of languor and oppression; anorexia; alternate feverish and chilly sensations; pulse low, and when I saw J. H., his skin was cold and clammy, (the general initial symptoms of the sick just now)." In the succeeding month commenced the fever above described.

I have taken a rapid glance at the primary character of the ailments in 1847. Did those of 1848 bear the same stamp? Not exactly; though still the resemblance was considerable; but the average of sickness was in my neighborhood much less. During the early months, the cases bore very much the same features, as in the latter months of the preceding year. But in May, intermittent fevers became common, in many cases, running into congestive remittents: the same may be said of July, the congestive character increasing in frequency and gravity. During this month, there were also some cases of Jaundice; and in the low grounds towards the mouth of the Bonpas Creek, some few cases of Typhoid Erysipelas. In Aug. the tendency to low congestive remittent still increased; accompanied in its own localities, with milksickness; the same may be said of Sept., the amount of sickness abating towards the latter end. Through Oct. Nov. and Dec., the cases were of a promiscuous character; with some few instances of Typhoid Pneumonia, towards the end of the year.

Now, through all this period, in all these various phases of disease, or disorder, as Armstrong, I think more correctly calls it—was there that similarity of symptoms, that would warrant the opinion I entertain of their common parentage? And here I would remark, that I have great doubts whether malarious effects are generally manifested, the season of the inception of the cause; as the greatest results are generally perceived in the middle months, while it is most probable, that the greatest elaboration would be towards autumn. We know, indeed, that the disease may lay dormant, till some exposure to a further depressing cause, as cold or fatigue, lays a spark on the train. Of this we have a remarkable example in the cases of the British soldiers, who returned apparently unscathed, from the Walcheren Island, where so many of their comrades had fallen victims to its malignant intermittents; and yet, some months after their return to a salubrious atmosphere had, by mere change of weather, the same disease developed. It has probably been remarked by others, as well as myself, that emigrants rarely become subject to endemic diseases, the first season they are here. A modern English writer, (Carbutt) in speaking of the exemption of Manchester from intermittents, observes:—"But do we never see this ague there in Manchester? oh yes; we see plenty of it. The poor Irish who go in the fall to assist in the harvest, in Lincolnshire, Cambridge, &c., come, many of them, to winter in Manchester; and the first east wind that blows in February or March, brings out the paroxysm of ague, which they had caught in the autumn; but which had lain undeveloped and unsuspected in the system, till aroused by such weather as we generally have in February and March." *Till* (he might have said) a call was made upon the vital energies of the system which had been sapped before.

Well, then, let us look back and sum up the evidence. In the early months we find but little peculiar: their diseases in fact belong to the group of winter, and it is not till about April or May, we must look for the starting point. Then we see Intermittents of mild character generally, merely exhibiting the want of nervous tone; but gradually progressing with the year, and the fresh obnoxious cause, aided by the debilitating effects of heat and fatigue, into a graver character. It is now we begin to meet the imperfect reaction, the protracted chill; the prostrate nervous energy: showing clearly that the being taken sick, as it is called, is but the bursting forth of the smouldering fire; for they have been long sick, and the visceral complications are the result of protracted causes.—

Question your patient: he will tell you he has not felt well for some time; he was quite unwell some weeks back, but got better; he has had bad taste in his mouth; irregular bowels; his appetite has often been capricious; but he felt well when at work. We all know how common is such a history, and yet with all this he thinks he was well, and he dates back his present attack to some cold, some getting wet, &c.

Let us look further. We have had cases of Milksick and Trembles, as it is called, with the long premonitory tires, as popularly styled, succeeded or accompanied by irregular appetite sometimes in excess, then entirely wanting; the fœtid breath; the irregular bowels; the pains in the back and limbs; and lastly, in most cases, the torpor of the bowels, often amounting to absolute constipation. In most cases, there is for a few days, an attempt at reaction or fever, but it fails, and then there is the cool skin; the burning at the epigastrium; the excess of acid, secreted by the stomach: the consequence, I suppose, at first, and afterwards the cause, of the gastric pain: the excessive thirst; and often again, the ravenous appetite; the oppressed pulse; the depressed nervous sensibility, as evidenced in most cases after the first few days, by the dilated pupils; the miserable indescribable restlessness, the common consequence of congestive oppression; the black, tarry, fœtid stools; or else the green scylabous ones, (when stools are obtained.)

In some of these cases, the symptoms so vague, that even the neighbors, with all the popular belief in its specific and distinctive character, will contest among themselves, the question of its identity; and yet its fraternity could not be doubted by the observant physician, if similarity of pathological derangement, and symptoms, grouped in like order, and only differing in degree, be the criteria in diagnosis.

I have described as occurring at the same time, or just preceding—but in different parts of the county—Congestive Remittent Fever, of a most grave character; with headache; pains in the limbs and back; vomiting; and in most cases, accompanying Diarrhœa, with epigastric and abdominal tenderness; and as the weather grew colder, Pneumonia. But what class of Pneumonia? Was it with high febrile excitement; pain; cough; hot skin, and flushed face? No, indeed: but a true typhoid disease, in a large proportion of the cases; with dry, dark tongue, and crusted teeth; Diarrhœa; feeble, quick pulse; and listless, yet restless feelings; and but little thoracic sign, but what the stethescope supplied.

In the succeeding year, 1848, we find pretty much the same record: the same classes of disease, appearing in the same localities. The following case, in Aug. and Sept., will illustrate in detail, the fevers of that year, classed by me, as congestive remittent. The location, a flat prairie, to the north east of a timbered swamp:

E. F., a girl of about 15; sanguine nervous temperament, was taken on Monday, 28th Aug., with fever and lassitude; no chill noticed; took some pills, which moved her bowels. I saw her on the 31st; at that time, the motions were dark and watery, with yellow shreds floating therein; tongue, broad, moist, and coated whitish yellow, with red points projecting through the coat, towards the apex; has had headache; is now dull and restless. On the night of the 29th was delirious; dropping asleep and waking up in great alarm; pulse soft and small; skin not hot; passes urine often, but in small quantities; has no appetite; not very thirsty, till last night. Quinine gr. vii.; Morphine gr. $\frac{1}{8}$; Blue Mass gr. v., every six hours; Sp'ts Ether Nit. Aqua Camph. equal parts; a table spoon full in water, every three hours.

1st Sept. Yesterday about 4 P. M., had a hot skin, which cooled down during the night; pulse now 72, soft and full; skin cool, and sweating; tongue moist, and coated whitish yellow; still some red points; there is a dry furred stripe, rather brown at the root, down the centre of the tongue, but not reaching to its point; slept pretty well nearly all day yesterday and last night; bowels not moved; urine more copious; face occasionally flushed; her eyes are natural; she is very susceptible to noise or talking, (perhaps caused by the Quinine;) has not eaten anything; not much thirst; says she has no pain anywhere. Quinine Sulph. Ex. Gentian aa. ℈j. in pills ix. three to be taken every six hours, with Blue Mass gr. v., continue Spirits Nitre and Aqua Camph.

2nd Sept. Is better; skin cool; pulse 70, small and weak; tongue clean and moist; no headache; no pain; bowels open; some scybala; slept well last night; has taken some chicken broth; continue same treatment, with wine or beer.

4th Sept. She is mending rapidly. ℞. Calomel gr. x., Jalap gr. x., and then resume the quinine pills, as before.—Dismissed.

This was a very mild and favorable case, yielding readily to treatment: I select it for its brevity. I would furnish many more, some of much greater severity; some mixed up of these symptoms, and those of the supposed milksickness, but I have already extended this paper too far.

These were diseases, in my belief, depending on Malaria for cause or color; and here I would wish to be understood, that I intend not to argue, that all diseases are so caused, nor even that all the cases that I have refered to, were wholly of that character. But that in all, the peculiar grades they took, when once developed, was dependent on the previous depressed nervous condition; the effect of Malaria. In the same way as the hysterical constitution, or the acquired diathesis of the habitual inebriate, modifies the character of every ailment to which such individuals may become subject.

I consider Malaria to be a depressent poison; the peculiar effect of which is to obtund or crush (in proportion to its concentration, and the constitutional resisting power of each individual,) the nervous system of those exposed sufficiently long to its action; as we see manifested in the oppression and Malaise, so generally experienced by persons for days or weeks previous to the attack of sickness, and as shown in the prostration, and insensibility to the stimulus of medicines, &c., in many bad cases of fever; and as we see in its more exaggerated form, in those fearful cases, where, as in a person asphyxiated, they sink beneath the cause, without nature appearing even to make a struggle.

In the following remarks, then, I assume the agency of Malaria as proved; and shall only now briefly point out the mode and channel, by which, I conceive, its fearful powers are brought to bear upon the system. In order more effectually to convey my explanation of its effects, I would recall to your attention the fact, and one which I think cannot be too familiar to our minds, of the division of the nervous system as defined by Bichat, into the nerves of relation or animal life, and nerves of nutrition or organic life. And it is to these last, that I think our attention is especially called, in connection with this subject; admitted as it now is, by Physiologists, that from the ganglionic system are derived those nerves lining the blood vessels; and therefore, receiving the first impression from all elements circulating in the blood, and also controling nutrition, secretion and absorption. Malaria we have assumed to be gaseous; and though capable probably, of being to some extent absorbed through the skin, to our clothed bodies at least, its introduction must be clearly by the lungs; and let its constituents be what they may, must, like the oxygen of the air, enter the blood by endosmosis: thus in a moment is the whole nervous tissue, lining the vast area of the vascular system, subjected to its influence; which, if of concentrated force, and the resisting power relatively small, crushes it at once, as in some rare instances we see. Or acting gradually and slowly from its diluted state, paralyses by degrees or perverts, that vital energy, upon which depends the healthy functions of all the organs of secretion, or absorption; for what, after all, is secretion? what is absorption but vascular action?—What are the organs of secretion or absorption, but certain congeries of vessels specially circumstanced, and specially endowed for certain and specific departments of the same function? But is this prostrated condition of these functions, the whole amount of the mischief? Unfortunately not! This deranged condition of the several organs designed to purify or repair, leaves the circulating fluid, from neglect of its natural guardians, loaded with elements which should have been removed, and therefore, unfit for healthy nutrition of the tissues; and thus the torpor of function of one organ, may throw corruption and derangement through the whole. Thence results either excitement of function primarily, or by reaction, as in the excessive secretion of Dysentery or Diarrhœa, when endemic or epidemic, or accompanying or complicating fever, and perhaps we might even say in Cholera: or producing torpor, palsy of the intestinal muscular fibres, as exhibited in the so called milksickness, and the character of which palsy, is so peculiarly illustrated to us all in the constipation of lead colic; a disease receiving its special cause also, either by cutaneous or gastric absorption, or by inspiration; but from either mode producing the same results. But to return to our text. This same cause, manifests its action in either excessive secretion or suppressed secretion; in depressed capillary action and decreased capillary area, as we see during a chill, and the engorgement of the internal organs as its consequence, as we know occurs in the spleen and liver, and doubtless also in the network of veins surrounding the Theca Vertebralis, and, I believe, the cause therefrom of those dorsal pains, so constantly accompanying the first periods of our fevers, as also supplying us with a ready explanation of the origin of those anomalous pains in the limbs, of which our patients under such circumstances, make such bitter complaints. Upon what other view can we explain the strange symptoms we have most of us occasionally met with, of pain in the great toe or heel, and there only; a symptom said to have been peculiar to a disease known and dreaded through this country, many years ago, called by the people, Cold Plague, very fatal, little understood, and doubtless a peculiar shade of Congestive fever.

Now, then, I think I have shown that certain painful symptoms, common in many of our endemic diseases, are readily accounted for on clear pathological principles. Let us see what Dr. Bell, the great contemner of Malaria, says in reference to the production of local effects from general causes. In showing the one sided reasoning of the followers of Broussais, he has the following:—"In the first place, let us inquire whether any cause acting on the whole economy, is capable of producing local disease. Now I believe it is quite certain that such is the fact, and that we may have first a morbid condition of the whole system, and consequent on this, nervous local lesions. Several continental pathologists, but in particular, M.M. Gaspard and Majendie, have shown by repeated experiments, that we can produce all the phenomena of Typhus in the lower animals, by introducing putrid substances into the system. These gentlemen injected putrid substances into the veins of animals and applied them to the surface of wounds; and in every case where these experiments were performed, they observed that the animals became ill; had langour; loss of appetite; thirst; prostration; in fact all the symptoms of bad typhus, and in case of death, they exhibited local lesions, corresponding with those we meet with in the human subject in fever." Here, then, is admitted the capability of agents acting on the general organism to produce local effects; the accompanying symptoms too, of which, closely correspond with those we have sketched as resulting from Malaria. But surely I have said enough. That certain agents of a gaseous form, can, when inhaled, produce all these effects is well shown in the following extract from Christison's work on Poisons:—"In March 1817, a number of miners at Lead hills, were violently affected, and some killed, in consequence, as was supposed, of the smoke of one of the steam engines having escaped into the way gates, and contaminated the air. The gas present was supposed to have been sulphurous acid gas with carbonic, both in a diluted state; some who attempted to pass through died immediately, some made their escape after some hours, but some remained till the air was so far purified, that their companions could descend to their aid. When Mr. Braid first saw them, they were running about frantic and furious, and striking all who came in their way; some ran off terrified, when any one approached them; some were singing; some praying, and others lay listless and insensible; many retired and vomited. In some the pulse was quick, in others slow; in many irregular, and *in all feeble;* all who could describe their complaints had violent headache; some Tenesmus, and a few Diarrhœa; many recovered in a few days."

But let it be remembered that in these cases the poisonous gas was inhaled at once, and not during a long period, in a state of great dilution, like a secret foe, working unseen, till the superstructure of our health is trembling to its base. That constant and long continued obstruction of the cutaneous functions, from cold, or damp, or other cause, may by suspending the proper depuration of the blood, cause it, after a time, to become so loaded with effete matters, that by the irritation it may produce from the excited vicarious action of other organs, and the depraved nutrition supplied to the body, may give rise to conditions, and symptoms of those conditions, in many points analogous to the effects of miasmatic agents, I do not dispute. But such would undoubtedly be cases of exception; similitudes, and not the rule, or the thing itself. The ordinary effects of vicissitudes of heat and moisture, we all know to be inflammatory diseases; such as simple acute Pleurisy; acute Pneumonia; Rheumatism; Inflammatory Erysipelas; Inflammatory Fever; Peritonitis, &c., &c., These are effects from sudden causes, acting on previously healthy bodies, and widely different, I conceive, would be our diagnosis, prognosis and treatment. In this relation, is, I think, one of the most important bearings of our subject Are we to consider all these cases in different districts, and on different soils, as alike? Are we to treat Pneumonia, as occurring in our low, swampy grounds, with the same means we would apply to the same disease in a hardy mountaineer? Is Erysipelas when epidemic to be treated in like manner, to the same disease, occurring in a healthy subject from some local irritation; or rather do we not know, that in certain spots, call them malarious or what you will; let the cause be bad air of one kind or another; our patients wear, not only in the pallor of their skins, and the size of their solid abdominal organs, but in the grade of their diseases, the stamp of their locality. In the words of Moore,

"The trail of the serpent is over them all."

The proverb says, tell me the company you keep, and I will tell you what you are. But I say, tell me where you live, and I will make a very close guess at the character of your disorders: and indeed, is it true, that full often, to look in a man's face alone, tells but too plainly the character of the soil he lives on, and the air he breathes.

